# CRITICAL ENGAGEMENT WITH AI: MAPPING ETHICAL PRINCIPLES TO APPLICATIONS IN AGING

**DOI:** 10.1093/geroni/igae098.0914

**Published:** 2024-12-31

**Authors:** Clara Berridge

**Affiliations:** University of Washington, Seattle, Washington, United States

## Abstract

Navigating the algorithmic age is difficult because of many unresolved ethical issues and policy gaps. A significant barrier to addressing ethical problems in AI and aging is that their scope is often restricted, leaving questions of power and social impact ill-defined. A contributing phenomenon is that people tend to take algorithmic output as objective, authoritative, and fair. In this way, ideas about objective data-based truth can convert questions that are ethical in nature into technical questions. Examples of questions with ethical aspects that are often not treated as such include which problems are prioritized for datification and how they are defined, which data are mobilized and sidelined, how data are collected and monetized, how representative data are, how older adults are involved in data collection, and how algorithmically mediated decision-making plays out with what differential impacts. Further, as technologies incorporate socially persuasive techniques, it is important to examine the ethical issues, such as implications for autonomy of misplaced trust with low awareness of these techniques. Recently, the broader discourse about ethics in AI that includes questions of power and societal impact has sharpened. The White House’s Blueprint for An AI Bill of Rights operationalized core principles that design, policy, and practice should uphold to protect people from potential harms. These principles are safe and effective systems, algorithmic discrimination protections, data privacy, notice and explanation of use of AI, and meaningful human alternatives. This presentation will discuss principles to promote ethical use of AI in tools and services for older adults.

